# Molecular Determinants of Uterine Receptivity: Comparison of Successful Implantation, Recurrent Miscarriage, and Recurrent Implantation Failure

**DOI:** 10.3390/ijms242417616

**Published:** 2023-12-18

**Authors:** Veronika Günther, Leila Allahqoli, Anupama Deenadayal-Mettler, Nicolai Maass, Liselotte Mettler, Georgios Gitas, Kristin Andresen, Melanie Schubert, Johannes Ackermann, Sören von Otte, Ibrahim Alkatout

**Affiliations:** 1 Department of Obstetrics and Gynecology, University Hospitals Schleswig-Holstein, Campus Kiel, Arnold-Heller-Strasse 3 (House C), 24105 Kiel, Germany; veronika.guenther@uksh.de (V.G.);; 2 University Fertility Center, Ambulanzzentrum of University Hospitals Schleswig-Holstein, Campus Kiel, Arnold-Heller-Strasse 3 (House C), 24105 Kiel, Germany; 3 School of Public Health, Iran University of Medical Sciences (IUMS), Tehran 14535, Iran; 4 Private Gynecologic Practice, Chrisostomou Smirnis 11Β, 54622 Thessaloniki, Greece

**Keywords:** implantation, uterine receptivity, cytokines, recurrent implantation failure, recurrent pregnancy loss

## Abstract

Embryo implantation is one of the most remarkable phenomena in human reproduction and is not yet fully understood. Proper endometrial function as well as a dynamic interaction between the endometrium itself and the blastocyst—the so-called embryo–maternal dialog—are necessary for successful implantation. Several physiological and molecular processes are involved in the success of implantation. This review describes estrogen, progesterone and their receptors, as well as the role of the cytokines interleukin (IL)-6, IL-8, leukemia inhibitory factor (LIF), IL-11, IL-1, and the glycoprotein glycodelin in successful implantation, in cases of recurrent implantation failure (RIF) and in cases of recurrent pregnancy loss (RPL). Are there differences at the molecular level underlying RIF or RPL? Since implantation has already taken place in the case of RPL, it is conceivable that different molecular biological baseline situations underlie the respective problems.

## 1. Introduction

Embryo implantation is one of the most remarkable phenomena in human reproduction and is not yet fully understood. Proper endometrial function as well as a dynamic interaction between the endometrium and the blastocyst—the so-called embryo–maternal dialog—is necessary for successful implantation. Several physiological and molecular processes are responsible for this success. The time frame is the so-called window of implantation (WOI), which is the specific time slot during which the endometrium is of an appropriate structure and receptive for implantation. This period is usually between the 20th and the 24th days of the menstrual cycle, during the secretory phase [1]. The adhesion of the blastocyst occurs during the implantation phase. The adhesion molecules are the generic term for a whole family of molecules that fulfill functions in various areas of the reproductive system [2]. Cadherins, integrins, trophinin, and selectin are just a few examples of adhesion molecules, which are responsible for the adhesion and the required physical interaction between the endometrium and the blastocyst [2,3]. There might be cell–cell and cell–extracellular matrix interactions leading to a successful implantation [3]. The adhesion is followed by the invasion of trophoblast cells surrounded by an immune-modulated environment, tolerating the embryo even though half of the genes are foreign to the maternal organism because they are of paternal origin [4,5]. Only a downregulation of the maternal immune system, which affects different cell populations such as the natural killer cells or T helper cells, makes successful implantation possible [5]. Pinopodes are small microvilli on the apical surface of the epithelial cells that appear on the 20th and 21st days of the cycle (may vary by 5 days) and characterize endometrial receptivity [1]. However, this receptivity is complex; it is a result of several physiological and molecular mechanisms [6].

In the following, implantation and its molecular processes, and especially the involved cytokines, will be described in detail in three different situations: successful implantation, recurrent implantation failure (RIF), and recurrent pregnancy loss (RPL). RIF is defined as the failure to achieve a pregnancy after three or more consecutive in vitro attempts, with the transfer of at least four high-quality embryos in three fresh or frozen cycles [4,7]. RPL is defined as three consecutive pregnancy losses prior to 20 weeks of gestation. The American Society for Reproductive Medicine defines RPL as the failure to achieve pregnancy even after two or more pregnancy losses with clinical (ultrasonography or histopathology) evidence of pregnancy [4,8]. There are a number of causes that are discussed to be associated with RIF or RPL, such as anatomical factors, chromosomal causes, or thrombophilia. Table 1 provides an overview of these possible factors.

Concerning the implantation process, there are several cytokines involved, such as: IL-6, IL-8, leukemia inhibitory factor, IL-11, IL-1, and the glycoprotein glycodelin. Accordingly, an imbalance could occur in the event of RIF or RPL. Are there differences at the molecular level underlying RIF or RPL? Since implantation has already taken place in the case of RPL, it is conceivable that different baseline states of molecular biology underlie the respective problems. In the following, we will first describe the molecular factors of successful implantation and early pregnancy development, and then explain the respective molecular situation in RIF and RPL. By accurately analyzing the particular pathomechanism at the molecular level, it may be possible to develop strategies that will help, in the clinical setting, to devise a suitable therapeutic approach for the individual patient with RIF or RPL and achieve a successful pregnancy. Furthermore, this review will focus on estrogen, progesterone, and their receptors but will not take the ovarian function, including FSH, LH, and AMH and their relation to pregnancy, into account.

## 2. Estrogen, Progesterone, and Their Receptors

Estrogen and progesterone and their respective receptors are among the first factors that influence the uterus and prepare the uterine environment for implantation. During the menstrual cycle, the estrogen level rises with the growing follicle, reaches its peak during ovulation, and then decreases. Alongside the falling estrogen level, progesterone rises and is the leading hormone during the luteal phase, inducing decidualization and opening the window of implantation (WOI) [12]. If pregnancy occurs, the progesterone level remains elevated. The WIO is characterized by balanced estrogen and progesterone levels, leading to a proper proliferated and transformed endometrium for blastocyst invasion. Any disruptions of this well-balanced system results in a failed implantation of the blastocyst.

However, not only estrogen and progesterone levels are important for implantation; their receptors also appear to play an important role in this process. Estrogen receptors exist in two forms: ERα and ERβ. Both are expressed in the endometrium but have different functions. Studies on knockout mice have addressed these respective functions. ERα knockout mice show endometrial hypoplasia and are infertile. Thus, ERα appears to be essential for implantation [13]. In contrast, ERβ knockout mice have a normal endometrium and are fertile, suggesting that ERβ is involved in other aspects of endometrial function [14]. During the proliferative phase, estrogen (through ERα) causes the progesterone receptor (PR) in endometrial cells to induce progesterone responsiveness during the luteal phase. As a negative feedback, progesterone inhibits ERα expression for correct endometrial function. The effects of progesterone in endometrial cells are mediated by the progesterone receptor, which exists in two isoform: PR-A and PR-B. Progesterone effects mediated by PR-A appear to be responsible for correct implantation, pregnancy, and parturition. This has been shown in PR-B knockout mice. The effect of progesterone is mediated by PR-B in PR-A knockout mice, and, here, scientists have found endometrial hyperplasia, inflammation, and the absence of decidualization of the endometrium [15]. Knockout mice for both PR-A and PR-B are infertile, showing severely reduced or no further ovulation, uterine hyperplasia, the absence of decidualization, severely limited mammary gland development, and impaired sexual behavior [16]. These study data can be extrapolated to humans, demonstrating the importance of progesterone receptors and their different functions in fertility.

### 2.1. Estrogen and RPL

Rising estrogen levels during follicle growth are responsible for endometrial proliferation, myometrium thickness, and increased blood supply: all of these are factors for successful implantation. Estradiol levels during early pregnancy can reflect the quality of the dominant follicle and the function of the corpus luteum as well as help in maintaining the corpus luteum [17]. Furthermore, estradiol appears to be an important factor in preserving early pregnancy [18]. In the 4 to 8 weeks of pregnancy, a positive correlation was found between serum estradiol level and gestational age. Serum estradiol levels were significantly lower in pregnant women with abortion than in those with normal pregnancy [17]. Deng et al. analyzed serum levels of estradiol, progesterone, and human chorionic gonadotropin (hCG) in 165 women during 9 weeks of gestation. A total of 71 women had a miscarriage, whereas 94 women had a normal pregnancy. Low levels of estradiol, progesterone, and hCG were associated with a miscarriage in the first trimester. The authors concluded that estradiol and progesterone or estradiol alone at 7–9 weeks and hCG or progesterone in combination with estradiol at 5–6 weeks of gestation can be used to predict miscarriage [19].

### 2.2. Progesterone and RPL

A deficiency of progesterone itself and an aberrant PR-mediated signaling may play a role in RPL. As mentioned above, progesterone induces decidualization, opens the WOI, and maintains pregnancy. A progesterone deficiency and a shortened luteal phase may result in suboptimal endometrial development, which has been associated with RPL [20]. On the other hand, it has been stated that a progesterone deficiency is rather an expression of a previously insufficient follicular phase, which should be optimized in advance. While the effect of progesterone through PRs plays an important role in implantation and the maintenance of pregnancy, progesterone supplementation in patients with sporadic miscarriage does not appear to improve the outcome of pregnancy [20]. In a randomized controlled trial in the UK, the authors analyzed 4153 women with vaginal bleeding during early pregnancy, who were receiving either progesterone or placebo. The progesterone therapy did not result in a significantly higher rate of live births among women with threatened miscarriage overall, but women with early-pregnancy bleeding and previous miscarriages in the past had a higher number of live births [21]. The authors of a meta-analysis conducted in 2021 reached similar conclusions: again, vaginal micronized progesterone resulted in a higher live birth rate in female patients with a history of vaginal bleeding and miscarriage(s) in the past [22].

### 2.3. Estrogen, Progesterone, and RIF

Estrogen plays a crucial role in endometrial receptivity due to the initiation of paracrine or autocrine signaling [23]. Higher or lower estrogen levels in the periconceptional period lead to lower pregnancy rates, as well as in natural cycles and also in assisted reproductive technology (ART) cycles. In naturally conceived pregnancies, low estrogen concentrations are associated with non-conception cycles [23]. On the other hand, excessive supraphysiologic estrogen levels at the time of the luteinizing hormone (LH) peak correlate with lower live birth rates and a higher risk of pregnancy complications [23]. In very early pregnancy, estrogen plays an important role in placentation by modulating the angiogenic factor expression and causing an immune-tolerant environment by influencing the concentration of a uterine natural killer and T helper cells, which are important for implantation [23].

In women with RIF undergoing ART treatment, hormonal stimulation leads to supraphysiological estrogen levels and possibly to a premature increase in progesterone. The endometrium is exposed to the premature impact of progesterone, which causes asynchronicity between the embryo and the endometrium, and leads to suboptimal implantation conditions [1]. A premature rise in progesterone before ovulation can be detected by a laboratory analysis. Consequently, no embryo transfer but a freezing of all fertilized eggs should take place.

Furthermore, a decrease in ER and a polymorphism in estrogen receptor 1 was noticed in patients with a recurrent implantation failure [24,25].

## 3. Molecular and Cellular Events Involved in Successful Embryo Implantation

Interleukins, interferons, chemokines, and numerous other mediators can be summarized as cytokines which are produced by different effector cells and play an important role in the human innate and adaptive immune systems [26]. Based on their properties, cytokines may be pro- or anti-inflammatory, as shown in Table 2. The fragile balance and complicated relationship of cytokines are caused by their overlapping biological activities; any alteration of one cytokine is likely to affect the others.

The interleukin (IL)-6 family is a class of cytokines consisting of IL-6, IL-11, ciliary neurotrophic factor, leukemia inhibitor factor (LIF), oncostatin M, cardiotrophin 1, cardiotrophin-like cytokine, and IL-27. They belong to one family because the receptor complex of each cytokine contains two (IL-6 and IL-11) or one molecule (all other cytokines) of the signaling receptor subunit gp130 [27]. In this review, we will focus on IL-6, LIF, and IL-11 because they play key roles in the implantation of the embryo [1]. Furthermore, we will analyze IL-8, IL-1, glycodelin, and their roles in successful implantation, as well as RPL and RIF.

**Figure 1:**
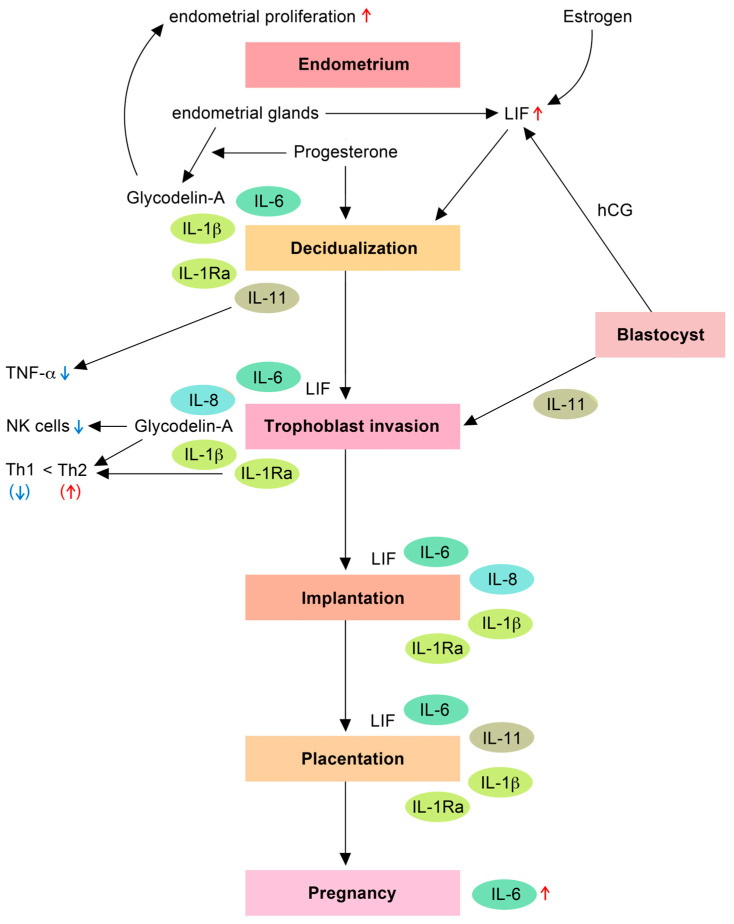
**Function of hormones and molecular determinants in implantation and early pregnancy.** Progesterone, LIF, IL-6, IL-11, IL-1β, and IL-1Ra are involved in regulating decidualization. LIF is increased under the influence of estrogen and hCG. LIF, IL-6, IL-8, glycodelin-A, IL-1β, and IL-1Ra are responsible for trophoblast invasion. As regards shifts in the immune system, IL-11 reduces TNF-α, glycodelin-A lowers the levels of NK cells, while glycodelin-A, IL-1β, and IL-1Ra are responsible for the shift within Th cells. LIF, IL-6, IL-8, IL-1β, and IL-1Ra are associated with embryo implantation. Furthermore, LIF, IL-6, IL-11, IL-1β, and IL-1Ra participate in placentation. Abbreviations: LIF (leukemia inhibitory factor), TNF-α (tumor necrosis factor-α), NK cells (natural killer cells), Th1/2 (type 1 T helper cells and type 2 T helper cells), IL-1Ra (IL-1 receptor antagonist), ↓ (down-regulated), ↑ (up-regulated).

Figure 1 summarizes the above-mentioned molecular determinants and their functions in regular implantation and pregnancy. These components will be described in the following.

**IL-6** is a pleiotropic cytokine and is responsible for many physiological processes. Its role in the immune response, inflammation, and metabolic regulation is known. However, it is also indispensable for the development of early pregnancy [28]. IL-6 is synthesized by macrophages, fibroblasts, epithelial cells, and placental trophoblasts, and it is found in high concentrations in the luteal phase, especially in the receptive window. It is responsible for the development of the placenta and pregnancy itself by regulation of trophoblast invasion and spiral artery remodeling [29]. IL-6 interacts by binding to its receptors and subsequently activating the Janus kinase/signal transducer and activator of the transcription (JAK/STAT) pathway [28]. The classical signal transduction of IL-6 is induced by the binding of IL-6 to its specific membrane IL-6α-receptor (IL-6R). This pathway is confined to a few tissues because of the restricted expression of IL-6R [30].

In the first trimester of pregnancy, different cell populations of the uteroplacental tissues (decidual stromal cells and different populations of immune cells of the decidua and syncytiotrophoblast, extravillous trophoblast, and cytotrophoblast cells of the placenta) express IL-6 [1]. IL-6 expression increases with gestational age [1].

**IL-8** is a proinflammatory chemokine produced by immune cells and other cells under inflammatory conditions, and it is responsible for the attraction of neutrophils in cases of inflammation, monocyte-macrophage growth and their differentiation, endothelial cell survival, proliferation, and angiogenesis [31,32,33]. Furthermore, IL-8 contributes to endometrial receptivity and maintains the dialog between the embryo and the human endometrium during implantation [34]. The upregulation of endometrial IL-8 mRNA occurs in the receptive phase of the menstrual cycle and also in the presence of an embryo [35]. IL-8 appears to stimulate progesterone secretion in order to maintain pregnancy [36].

### 3.1. IL-6, IL-8, and RPL

Several studies have shown a higher risk of pregnancy loss in cases of inadequate expression of IL-6 and IL-8 in the feto–maternal interface [37,38,39]. Interestingly, IL-6/IL-8 levels can be too low or too high, which could be responsible for sporadic abortion or recurrent pregnancy loss, respectively. In cases of sporadic abortion, decidual macrophages and decidual natural killer cells produce less IL-6 and IL-8 compared to the corresponding cells in normal pregnancy, which leads to suboptimal IL-6 and IL-8 levels and results in inadequate trophoblast invasion and spiral artery remodeling, and, thus, a higher risk of early pregnancy loss [37].

On the other hand, increased levels of IL-6 and IL-8 appear to be related to RPL [39]. Increased IL-6 and IL-8 levels in decidual tissues indicate a pro-inflammatory state at the feto–maternal interface, which could be disturbing to the implanted embryo and lead to an abortion. Previous findings have indicated that both insufficient and excessive levels of IL-6/IL-8 disturb the inflammatory network at the feto–maternal interface, which may compromise the pregnancy. Furthermore, differences in the expression profile of IL-6 and IL-8 in reproductive tissues between spontaneous pregnancy loss and RPL support the hypothesis that these complications may have a completely different pathogenetic background [28,40].

### 3.2. IL-6 and RIF

A small number of studies with limited case numbers have been focused on the relationship between cytokines and RIF [41,42,43].

Liang et al. analyzed the balance state of pro- and anti-inflammatory cytokines in the plasma of patients with RIF [41]. A total of 34 patients with RIF and 25 women with successful pregnancies were included. The interferon (IFN)-γ, IL-1β, IL-6, and IL-4 concentrations were higher, whereas the transforming growth factor (TGF)-β1 concentration was lower in the RIF group than in the control group [41].

Ozgu-Erdinc et al. reported different data regarding IL-6 and pregnancy outcome. In 129 patients, the authors correlated high-sensitivity C-reactive protein and IL-6 with pregnancy outcomes after in vitro fertilization (IVF)/intracytoplasmic sperm injection (ICSI). Serum levels were measured at the beginning of the IVF/ICSI cycle. No differences in C-reactive protein or IL-6 concentrations were seen on comparing implantation/no implantation, clinical pregnancy, miscarriage, and live birth [43].

### 3.3. IL-6, IL-8, and RIF

A study group in Beijing analyzed inflammatory cytokine levels in serum samples of women undergoing IVF [42]. A total of 84 women were included, of whom 46 conceived and 38 did not. Serum samples were taken on the second day of the menstrual cycle before the treatment cycle, and the inflammatory cytokines (interleukin-1β, interleukin-6, interleukin-8, and monocyte chemotactic protein-1) were measured. Women who did not become pregnant had significantly higher serum IL-8 levels than those who achieved a pregnancy [42]. Furthermore, a dose–response correlation between serum IL-8 levels and the risk of IVF-ET failure was noted, especially when the IL-8 concentration was >11.2 pg/mL [42].

**Leukemia inhibitory factor** (LIF) also belongs to the IL-6 family and is a pleiotropic cytokine. IL-6 in the uterus activates the Janus kinase (JAK)-signal transducer and activator of the transcription protein (STAT) pathway, and, therefore, phosphorylates STAT3, whose activation is necessary for implantation [15,44,45]. LIF is produced by the endometrial glands in order to make the endometrium receptive for blastocyst attachment [46]. The highest concentrations are registered during blastocyst formation immediately before implantation [47]. The rising estrogen levels during the menstrual cycle may be a stimulator of LIF expression [48]. LIF secretion is influenced by human chorionic gonadotropin as well as by the male seminal fluid [49,50]. Additionally, LIF is responsible for the decidualization of the stroma for implantation and, finally, for placenta development. During embryo implantation, LIF maintains the development of pinopodes and is, thus, responsible for embryo attachment, which is followed by invasion [51]. Furthermore, LIF is important for the formation of maternal decidua and trophoblast giant cells, as well as for the interaction of feto–maternal blood vessels [46].

### 3.4. LIF and RPL

Again, a small number of studies with limited case numbers have addressed the relationship between LIF and RPL. A case–control study comprised 30 fertile women and 30 suffering from RPL. Endometrium samples were taken in order to investigate the expression of LIF. The authors found a statistically higher mRNA expression of LIF in women with RPL [52].

Xu et al. came to a different conclusion [53]: the authors analyzed endometrial biopsy samples from women with RPL and compared the expression of LIF, integrin-ß3, and mucin-1, as well as the pinopode morphology, with those of normal fertile women. No differences were seen between the two groups with regard to any of the studied parameters [53].

A further recent study from Iran analyzed the role of LIF and inflammatory cytokines in women with RPL compared to a control group of fertile women [54]. Gene expression levels of LIF, tumor necrosis factor-alpha (TNF-α), and interleukin-17 were measured in peripheral blood. The mRNA levels of LIF were significantly lower in women with RPL compared to the fertile control group (*p* = 0.003) [54]. Regarding the other cytokines, no significant difference was seen between the two groups (*p* ≥ 0.05) [54].

LIF is regarded as a characteristic biomarker of endometrial receptivity. High expression of LIF appears to be an indicator of a receptive endometrium in fertile women. Nevertheless, the three studies mentioned above obtained different results concerning LIF concentrations in women with RPL. Higher, lower, and no differences in LIF expression were reported. Further studies with larger case numbers will be needed to make conclusive statements.

### 3.5. LIF and RIF

One of the first studies analyzing the correlation between LIF and RIF was performed in 1998 by Hambartsoumian [55]. Women with unexplained infertility and RIF were compared with fertile women as a control group. Endometrial samples were taken in the proliferative and secretory phases. Fertile women showed an elevation of LIF secretion in the secretory phase compared to the proliferative phase (2.2 times higher) [55]. In contrast, infertile women had no elevation of cytokine production. Furthermore, in infertile women with RIF, the LIF level fell in the secretory phase [55]. When the quantity of cytokine secretion was compared on the same day of the cycle between the two groups of women, LIF production in fertile women on days 18–21 of the menstrual cycle was 3.5 times higher than that in infertile women with RIF and 2.2 times higher than LIF production in women without RIF (*p* < 0.01 and *p* < 0.05, respectively) [55]. On days 8–11 of the cycle, the levels of LIF in these groups did not differ significantly. However, the distribution of cytokines varied in infertile women: the highest amplitude of variations was seen in patients with RIF. The author concluded that the majority of infertile women (especially those with RIF) have a dysregulation of LIF production in the endometrium during both the proliferative and the secretory phases of the cycle [55].

Wu et al. confirmed the above-mentioned data by analyzing the expression of LIF in endometrial samples of fertile women and women with RIF [56]. Fertile women had a moderate expression of LIF in the proliferative phase and a high expression of LIF in the secretory phase. In addition to the previous knowledge that LIF is decreased in the secretory phase, women with RIF had lower LIF levels in the proliferative phase compared to fertile women [56]. The data suggest that low LIF in the proliferation phase may also be partly responsible for repeated implantation failure.

Another interesting approach concerning the molecular causes of LIF downregulation in patients with RIF was described by a Chinese study group [57]. Women with a history of RIF showed abnormally increased levels of Krüppel-like factor 12 (KLF12), which in turn leads to reduced LIF expression with negative effects on endometrial receptivity, embryo adhesion, and implantation [57]. After analyzing the LIF gene sequence, the authors concluded that KL12 can directly suppress LIF expression, as it can bind to two KLF12 binding sites in the promoter of the LIF gene [57]. Interestingly, the authors also showed that KLF12 expression could be significantly reduced in vitro when Ishikawa cells overexpressing KLF12 are treated with medroxyprogesterone acetate (MPA). Consequently, progesterone may be a novel therapeutic approach for patients with RIF, as it acts upstream of both LIF and KLF12, inhibiting KLF12 expression and, thus, promoting LIF secretion [57,58].

**Interleukin-11** (IL-11) is a pleiotropic cytokine with anti-inflammatory functions [59]. It is produced by stromal and epithelial cells and maintains adequate decidualization [60]. The function of IL-11 is mediated by the IL-11 receptor (IL-11Rα), which is expressed by the luminal and glandular epithelia [60]. Unlike IL-11, which is at its highest concentration during decidualization, the receptor is not subject to cyclic variation. The different concentrations of IL-11 depend on its production, influenced by steroid hormones, and local factors such as relaxin and PGE_2_ [60,61]. Interestingly, there are differences between the stromal and epithelia cells: both express IL-11 but stromal cells produce the highest concentrations during decidualization. In contrast, epithelial cells express IL-11 primarily during the early secretory phase of the cycle. This suggests that epithelial and stromal IL-11 may play different roles in endometrial function [60,62].

In vitro studies have shown that IL-11 leads to a dose-dependent decrease in the production of the proinflammatory cytokine TNF-α. TNF-α production is highest in the secretory phase but then diminishes in early pregnancy. This reduction may be caused by a higher concentration of IL-11 [60]. In contrast, LIF and IL-6 were not found to affect TNF-α production [60].

IL-11Rα knockout mice are infertile, probably as a result of inadequate or absent decidualization and possibly over-invasiveness of the trophoblast [61]. This phenomenon can be explained by the downregulation of a metalloproteinase inhibitor [63].

The embryo itself produces IL-11 during trophoblast invasion, probably in order to influence the endometrium [59]. Interestingly, no receptors for IL-11 have been found so far in the human embryo [61].

### 3.6. IL-11 and RPL

A study group from the UK analyzed the endometrial peri-implantation biopsies of fertile women and women with RPL [64]. Both groups showed increased expression of IL-11 and IL-11Rα in epithelial cells compared to stromal cells [64]. Furthermore, there was a significant reduction in epithelial cell IL-11, but not stromal cell IL-11, expression in the endometrium of RPL women compared to fertile women. The expression of IL-11Rα protein did not differ between the two groups [64].

### 3.7. IL-11 and RIF

Karpovich et al. analyzed endometrial stroma cells and their IL-11 expression in women with primary infertility and compared them with a fertile control group [65]. The authors showed that the decidualization of endometrial stromal cells derived from women with primary infertility is defective, and that the production of IL-11 is compromised in these cells [65]. No differences were seen between the two groups in the expression of IL-11Rα in decidualized stromal cells, maintaining the hypothesis that the modulation of IL-11 expression, and not its receptor, is important in controlling the process of decidualization [65]. Thus, defects in endometrial IL-11 signaling may be associated with RIF.

Different results were reported by Sabry et al. The authors obtained endometrial tissue samples of women undergoing IVF and correlated IL-11 expression with pregnancy rates. No differences in gene expression levels of IL-11 and IL-11Rα were seen in women with implantation vs. non-implantation after IVF [66].

The **interleukin-1 (IL-1)** system consists of different components, including IL-1α, IL-1β, IL-1 receptor type 1 (IL-1R1), IL-1 receptor type 2 (decoy receptor, IL-1R2), IL-1 receptor accessory protein, and IL-1 receptor antagonist (IL-1Ra) [67]. IL-1α and IL-1β have proinflammatory properties and appear to play the most important roles in the development of pregnancy [59]. Unlike IL-1α, which is constitutively expressed, IL-1β is inducible [67]. The activity of IL-1β is regulated by IL-1Ra [59]. IL-1β is also an immune system modulator and is produced by dendritic cells, blood monocytes, T cells, and tissue macrophages [67]. With high affinity, IL-1Ra binds to IL-1R1 and, thus, inhibits IL-1α or IL-1β activity [68]. The extent of inflammation is influenced by the levels of circulating IL-1β, IL-1Ra, and IL-1R2 [69]. The expression of the components of the IL-1 system differs during the menstrual cycle, implantation, and early pregnancy [67]. Several studies have shown that the IL-1 system—especially IL-1β and IL-1—are responsible for the embryo–maternal dialog and the immunological shift that is necessary for the successful implantation of the blastocyst. The tissue of the fallopian tubes and myometrial smooth muscle synthesize IL-1β during pregnancy [70]. IL-1Ra has been found in the maternal, fetal, and amniotic fluid compartments, especially the decidua, and increases with rising gestational age in fetal and maternal plasma [71]. IL-1β plays an important role in the decidualization of stromal cells and in successful blastocyst implantation. IL-1β is produced by cytotrophoblast cells from the placenta of the first trimester, while lower expressions are seen in cultures from second- and third-trimester placenta [67].

In addition to its function in pregnancy, IL-1β influences the coagulation system by increasing the expression of several proteins that in turn increase platelet stability and raise the risk of thrombosis. This pro-thrombotic state with a higher risk of microthrombi during early pregnancy may be the reason for miscarriages [72].

### 3.8. IL-1 and RPL

The immune system plays an important role in implantation and early pregnancy [4]. The T helper (Th) cell populations and their corresponding cytokines have different, even contradictory, functions: whereas type 1 T helper cells (Th1) (including interferon-γ, IL-2, TNF-α) seem to disrupt pregnancy, type 2 T helper cells (Th2) (including IL-4, IL-5, IL-6, IL-10, IL-13) support and maintain the development of early pregnancy [4]. Women with RPL had increased levels of Th1 and low concentrations of Th2 [73]. IL-1β can induce Th2 responses in humans [67,74]. Furthermore, low levels of IL-1β and IL-6 mRNA have been found in the endometrium of women with RPL [67,75].

Löb et al. analyzed IL-1β levels in the decidua and obtained different results. Patients with spontaneous and recurrent miscarriages had a significantly higher expression of IL-1β than the control group with healthy pregnancies [76].

In addition, the IL-1 system appears to influence the outcome of pregnancy in women with the antiphospholipid syndrome. The latter is an autoimmune disease with, inter alia, a hypercoagulable state leading to thrombosis. The patients suffer from RPL and have elevated antiphospholipid antibodies [77]. The activation of inflammasome as well as secretion of IL-1β seem to increase inflammation, which has been proposed as one of the causes of RPL in the antiphospholipid syndrome [78].

### 3.9. IL-1 and RIF

Several studies have analyzed the relationship between IL-1 concentrations and pregnancy outcomes after IVF in order to describe RIF.

A retrospective investigation performed by Kreines et al. addressed IL-1β and IL-1Ra in maternal serum after IVF [79]. In healthy pregnancies, increasing IL-1β levels were registered over the duration of pregnancy. In cases of a negative pregnancy test or subsequent pregnancy loss, no increase was detected. Interestingly, women who initially had a positive pregnancy test and an abortion a few weeks later had undetectable IL-1β levels. In contrast, no significant data were registered in the serum IL1-Ra analysis [79].

A group from The Netherlands studied endometrial–embryonic interactions by taking endometrial secretions aspirated prior to embryo transfer from 210 women undergoing IVF [80]. Seventeen soluble cytokines, including IL-1β, were correlated with pregnancy outcomes using a multivariate logistic regression analysis. A significant negative association was registered between IL-1β levels and pregnancy outcome: clinical pregnancy was associated with lower levels of IL-1β. The predictive value of IL-1β (and TNF-alpha) for pregnancy was equivalent to, and in addition to, that of embryo quality [80].

These data are consistent with a previous report comparing women with RIF after IVF and controls, which demonstrated significantly higher IL-1β levels in endometrial flushings from women with implantation failure [81].

As mentioned earlier, women with recurrent miscarriage had significantly lower levels of IL-1β mRNA in the endometrium [75]. It is postulated that women with habitual abortion may have an ‘over-receptive’ endometrium that allows for the implantation of aneuploid embryos instead of leading to implantation failure, resulting in recurrent miscarriages of these embryos [82]. IL-1β levels appear to be related to achieving a clinical pregnancy rather than embryo implantation. This suggests that this cytokine may have a more important role in later stages of implantation rather than initial apposition and adhesion of the embryo [80].

### 3.10. Glycodelin

Glycodelin is a secretory protein responsible for different processes such as immunosuppression, fertilization, and implantation. Analogous to its functions, it has a temporospatial pattern of expression, primarily in the reproductive tract, where glycodelin is expressed in the mid-secretory phase. The peak occurs 10 days after ovulation and is regulated by progesterone. In addition to the secretory and decidualized endometrium, glycodelin is found in mammary glands as well as in bone marrow, ovaries, fallopian tubes, and seminal vesicles [83].

Depending on the respective attached glycosylation form, glycodelin may be divided into four glycoforms (glycodelin-A, -S, -F, and -C). The respective capital letter describes where each glycoform occurs. In the male reproductive tract, glycodelin-S is secreted by seminal vesicles to the seminal fluid. In the female genital tract, glycodelin-A is mainly expressed in endometrial epithelial cells and secreted into the uterine fluid and amniotic fluid. Granulosa cells secrete glycodelin-F into the follicular fluid and glycodelin-C is found in cumulus cells [83].

Glycodelin-A appears to play an important role in changing the immune system during early pregnancy by suppressing the cytotoxicity of natural killer cells, as well as shifting the concentration of Th-1 and Th-2 cells towards a Th-2-dominated area which is necessary for a successful implantation [84]. Glycodelin-A inhibits T cell proliferation, stimulates T cell apoptosis, and controls the immune response from B cells. Furthermore, glycodelin-A stimulates endometrial proliferation and maintains the attachment of the trophoblast as well as its hCG production [1].

### 3.11. Glycodelin and RPL 

Toth et al. analyzed the expression of glycodelin protein and mRNA in the endometrium and compared patients with a normal pregnancy to those who had an abortion and those who had a molar pregnancy [85]. Patients with an abortion had a significantly lower glycodelin expression than those with a healthy pregnancy. In contrast, women with a molar pregnancy showed an upregulation of glycodelin. Reduced glycodelin concentrations may lead to an overactive immune system that rejects the developing embryo and causes abortion. Furthermore, hCG appears to influence glycodelin expression because there are equal proportions between hCG and glycodelin in cases of molar pregnancy and in cases of abortion [85].

A German study group achieved similar results [86]. Glycodelin and the pregnancy zone protein in the decidua of patients with spontaneous and recurrent miscarriages were compared to the expression of these entities in the placental tissue of term infants. The spontaneous and the recurrent miscarriages groups had significantly lower glycodelin levels than controls [86].

### 3.12. Glycodelin and RIF

In order to investigate the correlation between glycodelin and RIF, endometrial cells and blood samples were taken during the window of implantation in women with RIF and from a control group of fertile women [87]. A significantly lower expression of glycodelin was measured in the blood and endometrial tissue of women with RIF compared with controls [87].

These data were confirmed by a Polish study group. The authors analyzed glycodelin expression in uterine fluid, as well as in serum and endometrium samples of women with RPL or RIF, and compared these with fertile controls [88]. In general, glycodelin levels in the serum were three times lower than those in the uterine fluid, and there were no differences between the three study groups [88]. Concerning the uterine fluid, the lowest glycodelin expression was noted in women with idiopathic infertility and infertile women with endometriosis. Compared with the fertile control group, these results were statistically significant (*p* < 0.0001) [88]. Women suffering from RPL had lower glycodelin concentrations than controls, but the difference did not achieve statistical significance [88].

In summary, glycodelin is an endometrial receptivity marker. The expression of glycodelin in the endometrium is mirrored in the uterine fluid and serum.

## 4. Conclusions

Successful pregnancy needs the correct interaction between (a) the individual hormones and their respective receptors. Furthermore, (b) adhesion molecules are responsible for the required physical interaction between the endometrium and the blastocyst, and, finally, (c) immune cells and cytokine signaling pathways act as mediators for the so-called embryo–maternal-dialogue. A dysregulation or inappropriate expression in one of these three sections may lead to an implantation failure or a pregnancy loss [3,89]. In the present review, we focused on the hormones estrogen and progesterone; the cytokines IL-6, IL-8, LIF, IL-11, and IL-1; and the glycoprotein glycodelin. We analyzed their respective functions during implantation and early pregnancy, and we compared the situations of normal pregnancy vs. recurrent pregnancy loss vs. recurrent implantation failure. The intention was to determine differences at the molecular level as to how cytokines behave at the time of implantation or in early pregnancy in order to draw conclusions about RIF and RPL. It would be conceivable that a cytokine is upregulated in the case of RIF and downregulated in the case of RPL.

As shown in Table 3, studies on this topic are very inhomogeneous. Even the investigation of RIF or RPL alone reveals diverse results regarding cytokine levels. Accordingly, no clear statement can be made as to how the individual cytokines behave in RIF or RPL.

This can be explained by the different populations and, in part, small sample sizes, the different timings of sample collection, and whether the cytokines were measured in the plasma, a whole tissue, or a specific cell population. The complicated relationship between cytokines and their overlapping biological activities means that the alteration of one cytokine is likely to affect others; this hinders the study of their roles in implantation, RIF, and RPL. Furthermore, large-scale investigations should address the role of cytokines during implantation and in different types of reproduction failure.

It is difficult to derive a direct clinical consequence or recommendation for action from the above-mentioned cytokine changes. In theory, it would be conceivable to use glucocorticoids or i.v.-immunoglobulins to reduce cytokines in general. However, this would be an undirected therapy and would affect all cytokines, including those that are already low or not elevated at all.

Targeted approaches to lower, for example, IL-6 levels, e.g., using tocilizumab, an IL-6 antagonist, are contraindicated in pregnancy and should be discontinued at least 3 months before conception [90].

Immunomodulatory therapy approaches to the treatment of RIF or RPL have already been analyzed in detail and are not included in the guideline due to their inhomogeneous data situation and their sometimes-severe side effects [91].

## Figures and Tables

**Table 1:** Possible causes for the occurrence of RIF or RPL [9,10,11].

Genetic factors	Chromosomal aberrations
	De novo chromosomal abnormalities of the embryo or certain gene polymorphisms
Anatomical factors	Uterine malformation
	Adhesions
	Polyp
	Submucosal fibroid
Microbiological factors	Bacterial vaginosis
Chronic endometritis	Factor V Leiden mutation
	Antithrombin deficiency
	Prothrombin mutation
	Protein C deficiency
	Protein S deficiency
Thrombophilia	Antiphospholipid syndrome
Endocrine disorders	Hyperthyroidism
	Hypothyroidism
	Diabetes mellitus
	Hyperandrogenemia
	Hyperprolactinemia
	PCOS
	Luteal phase defects
Immunological causes	Cytokine levels
	Natural killer cells (uterine and peripheral)
	T-helper cell type 1/type 2 quotient
	KIR receptors
	HLA antibodies
Lifestyle	Overweight
	Underweight
	Increased stress
	Alcohol consumption
	Nicotine consumption
Idiopathic	

**Table 2:** Pro- and anti-inflammatory cytokines.

Pro-inflammatory cytokines	IFN-γ
	IL-2
	TNF-α
	IL-1β
	IL-6
	IL-8
	IL-17
	IL-12
Anti-inflammatory cytokines	IL-4
	IL-5
	IL-9
	IL-10
	IL-11
	IL-13
	TGF-β1
	LIF

Abbreviations: INF-γ: interferon-γ, TNF-α: tumor necrosis factor-α, TGF-β1: transforming growth factor-β1.

**Table 3:** Molecular determinants, their function in normal pregnancy, and changes in RPL and RIF.

Hormone/Cytokine/Glycoprotein	Normal Pregnancy	RPL	RIF
Estrogen	Proliferation of endometrium, ERα: essential for implantation, ERβ: no restriction of fertility	Estrogen ↓ [17,19]	Estrogen ↓ [23,24] Estrogen ↑ → premature progesterone influence [23]
Progesterone	Inducing decidualization, opening the WOI PR-A (PR-B knock-out): implantation, pregnancy, parturation PR-B (PR-A knock-out): endometrial hyperplasia, inflammation, absence of decidualization	Progesterone ↓ [19]	Estrogen ↑ → premature progesterone influence, asynchronicity between endometrium and embryo [23]
IL-6	Luteal phase: high concentration, regulation of trophoblast invasion and spiral artery remodeling	Sporadic abortion: IL-6 ↓ [37] RPL: IL-6 ↑ [39]	IL-6 ↑ [41] IL-6 = [43] IL-6 = [42]
IL-8	Luteal phase and during embryo presentation: high concentration; stimulates progesterone secretion	Sporadic abortion: IL-8 ↓ [37] RPL: IL-8 ↑ [39]	IL-8 ↑ [42]
LIF	Receptivity of endometrium, decidualization, maintaining pinopodes, embryo attachment, implantation, placenta development	LIF ↑ (endometrial samples) [52] LIF = (endometrial samples) [53] LIF ↓ (serum) [54]	LIF ↓ (secretory phase), high amplitude (proliferative phase) [55] LIF ↓ (proliferative phase) [56] KLF12 ↑ → LIF ↓ [57]
IL-11	Produced by stromal and epithelial cells, during early secretory phase (epithelial cells), during decidualization (stromal cells), TNF-α ↓	IL-11 (epithelial cells) ↓ [64]	IL-11 (stromal cells) ↓ [65] IL-11 = [66]
IL-1	IL-1β and IL-1Ra are responsible for the embryo–maternal dialog and the immunological shift. The tissue of the fallopian tubes and the myometrial smooth muscle synthesize IL-1β during pregnancy, IL-1β ↑ during pregnancy, responsible for prothrombotic state	IL-1β ↓ [67,75] IL-1β ↑ [76] IL-1β ↑ and antiphospholipid syndrome [78]	IL-1β= no increase in contrast to healthy pregnancies, IL1-Ra = [79], IL-1 β ↑ [41,80,81]
Glycodelin	Glycodelin-A: shifting the immune system, endometrial proliferation, fertilization and implantation	Glycodelin-A ↓ [85,86]	Glycodelin-A ↓ [87,88]

Abbreviations: ER: estrogen receptor, PR: progesterone receptor, WOI: window of implantation, IL-1Ra: IL-1 receptor antagonist, KLF12: Krüppel-like factor 12, ↑: higher expression compared to regular implantation, =: no changes concerning the expression, ↓: lower expression compared to regular implantation, →: leading to.

## Data Availability

The data presented in this study are available on request from the corresponding author.

## Abbreviations

IL	interleukin
LIF	leukemia inhibitory factor
RIF	recurrent implantation failure
RPL	recurrent pregnancy loss
WOI	window of implantation
ER	estrogen receptor
PR	progesterone receptor
hCG	human chorionic gonadotropin
ART	assisted reproductive technology
LH	luteinizing hormone
JAK	Janus kinase
STAT	signal transducer and activator of transcription
IL-6R	IL-6α-receptor
INF-γ	interferon-γ
TGF	transforming growth factor
IVF	in vitro fertilization
ICSI	intracytoplasmic sperm injection
TNF	tumor necrosis factor
KLF12	Krüppel-like factor 12
IL-11Rα	IL-11 receptor
PGE2	prostaglandin E2
IL-1R1	IL-1 receptor type 1
IL-1R2	IL-1 receptor type 2
IL-1Ra	IL-1 receptor antagonist
Th1/2	Type 1 T helper cells and type 2 T helper cells
